# Serum ferritin as an adjunctive marker of dengue severity in the pediatric emergency department

**DOI:** 10.3389/fped.2026.1798539

**Published:** 2026-05-22

**Authors:** Ricardo Iramain, Alfredo Jara, Jorge Ortiz, Laura Cardozo, Rocío Morínigo, Lucía Franco

**Affiliations:** Pediatric Emergency Department, Faculty of Medical Sciences, Universidad Nacional de Asunción, San Lorenzo, Paraguay

**Keywords:** dengue, disease severity, emergency care, ferritin, hyperferritinemia

## Abstract

**Introduction:**

Hyperferritinemia has been described as a potential marker of disease severity in various conditions, including dengue infection, where it has been associated with worse clinical outcomes.

**Objective:**

To evaluate the utility of serum ferritin levels in discriminating dengue with warning signs or severe dengue.

**Materials and methods:**

This single-center prospective cohort study evaluated ferritin levels in relation to disease severity in patients aged 1 month to 17 years. Patients were classified into two groups: non-severe dengue (group 1, without warning signs) and dengue with warning signs or severe dengue (group 2). All cases were laboratory-confirmed by antigen detection or serology and were evaluated in the Pediatric Emergency Department between December 2023 and May 2024.

**Results:**

A total of 142 patients were included, of whom 42.2% were classified as group 1 and 57.8% as group 2. Median ferritin levels were significantly higher in group 2 compared with group 1 [841.5 ng/mL (IQR: 244–1,747) vs. 261.5 ng/mL (IQR: 130–645.5); *p* = 0.001]. A ferritin cutoff value of 382 ng/mL was associated with dengue with warning signs or severe dengue (39% vs. 14%; *p* < 0.001; RR: 4.0; 95% CI: 2.0–8.2). The area under the receiver operating characteristic (ROC) curve was 0.67 (95% CI: 0.57–0.75), with a sensitivity and specificity of 67%, a positive predictive value of 60%, and a negative predictive value of 73%. The positive and negative likelihood ratios were 2.0 and 0.49, respectively.

**Conclusion:**

Higher ferritin levels were observed in patients with dengue with warning signs or severe dengue. Although a cutoff value of ≥382 ng/mL was associated with this composite outcome, its discriminatory performance was limited. Ferritin may serve as an adjunctive biomarker but should not be used as a standalone predictor of disease severity in pediatric dengue.

## Introduction

Dengue fever remains a significant concern in areas where there is a risk of contracting the disease, with 390 million infections and 20,000 deaths reported annually (1). In Paraguay, dengue fever is an endemic disease characterized by inter-epidemic outbreaks (2, 3).

Clinical classification is based on the presence of warning signs and symptoms, which are related to complications of the disease (2, 4). Clinical management decisions depend on the presence or absence of these warning signs (5–7).

Ferritin is a cytosolic protein also present in other cellular compartments, such as the nucleus, mitochondria, and lysosomes. A small portion of ferritin is in the serum. Serum ferritin levels have been described as markers of acute and chronic inflammation in various infectious, rheumatological, hematological, and even malignant pathologies.

Hyperferritinemia is a potential marker of severity in various pathologies. Its increase may indicate activation of the monocyte-macrophage system, which is crucial in the inflammatory cytokine storm, although a direct causal role has also been described by activating various proinflammatory molecules (8, 9).

The association of ferritin with the severity of dengue has been studied, finding a link between the severity of the disease and increased serum values. Furthermore, an association with faster progression towards severe forms of dengue have been reported (10). Several studies have demonstrated the same association using different ferritin values as a cut-off point (11, 12).

Despite several reports associating ferritin with dengue severity, evidence in pediatric populations—particularly in Latin America—remains scarce, and cut-off values are inconsistent across studies. This study aims to contribute regional evidence to address this gap.

## Methodology

### Study design and population

This single-center prospective cohort study included patients aged 1 month to 17 years with a diagnosis of dengue fever confirmed by NS1 antigen testing or dengue IgM serology. Patients were evaluated in the Pediatric Emergency Department (PED) between December 2023 and May 2024.

### Ethical approval and consent

All data were handled confidentially. Informed consent was obtained from parents or legal guardians prior to enrollment. The study adhered to the principles of autonomy, beneficence, non-maleficence, and justice in accordance with the Declaration of Helsinki. Ethical approval was granted by the Institutional Research Directorate (Resolution No. 399) and the Institutional Ethics Committee (CEI No. 74).

### Exclusion criteria

Patients with a clinical diagnosis of dengue without laboratory confirmation were excluded, as well as those with coinfections (e.g., COVID-19 or other pathogens), concomitant bacterial sepsis or septic shock, immunosuppression, severe malnutrition, or comorbidities associated with elevated ferritin levels. These included chronic liver disease, chronic kidney disease, autoimmune diseases (such as systemic lupus erythematosus and rheumatoid arthritis), and hematologic disorders (9).

### Outcomes and procedures

Dengue severity was classified according to the Pan American Health Organization (PAHO) guidelines (2), based on the presence of warning signs indicative of increased capillary permeability and progression to the critical phase of the disease.

For the purposes of this study, patients were categorized into two groups:

Group 1: Non-severe dengue (without warning signs)
Group 2: Dengue with warning signs or severe dengue

Ferritin levels were measured between days 3 and 4 of illness in patients with non-severe dengue, corresponding to the expected critical phase. In patients with warning signs or severe dengue, ferritin was measured at hospital admission to minimize selection bias.

Clinical and laboratory data were recorded using a standardized data collection form, including diagnostic tests and clinical outcomes.

### Variables

The variables analyzed included demographic characteristics (age and sex), clinical severity group, presence of comorbidities, warning signs (persistent vomiting, severe and continuous abdominal pain, mucosal bleeding, lethargy, hemoconcentration, and thrombocytopenia), vital signs (heart rate, systolic and diastolic blood pressure, pulse pressure, and age-adjusted hypotension), laboratory parameters (ferritin levels), complementary studies (abdominal and pleural ultrasound), and management variables (outpatient care, hospitalization, length of stay, PICU admission, and need for inotropic support). Treatment variables included fluid resuscitation regimens and the number of fluid boluses administered.

### Statistical analysis

Descriptive statistics were used. Categorical variables were expressed as frequencies and percentages, while continuous variables with normal distribution were presented as mean ± standard deviation. Non-normally distributed variables were reported as median and interquartile range (IQR).

Comparisons between groups were performed using the Student's *t*-test or the Mann–Whitney *U* test for continuous variables, as appropriate. Categorical variables were compared using the chi-square test or Fisher's exact test. All tests were two-tailed, with a significance level set at *p* < 0.05. Relative risks (RR) with 95% confidence intervals (CI) were calculated.

Ferritin levels were considered the exposure variable, and the presence of dengue with warning signs or severe dengue was defined as the outcome. Receiver operating characteristic (ROC) curve analysis was used to determine the optimal ferritin cutoff value for predicting disease severity (Group 2 vs. Group 1).

A multivariable logistic regression model was constructed including ferritin levels and clinically relevant covariates (age, presence of comorbidities, day of illness at sampling, and selected clinical variables) to assess independent associations with the outcome.

### Sample size calculation

Sample size estimation was based on previous literature (13), which reported a prevalence of hyperferritinemia of 77.8% in patients with warning signs or severe dengue and 10.3% in non-severe dengue. Assuming a confidence level of 95% and a statistical power of 80%, the minimum required sample size was 14 patients per group.

## Results

**Figure 1:**
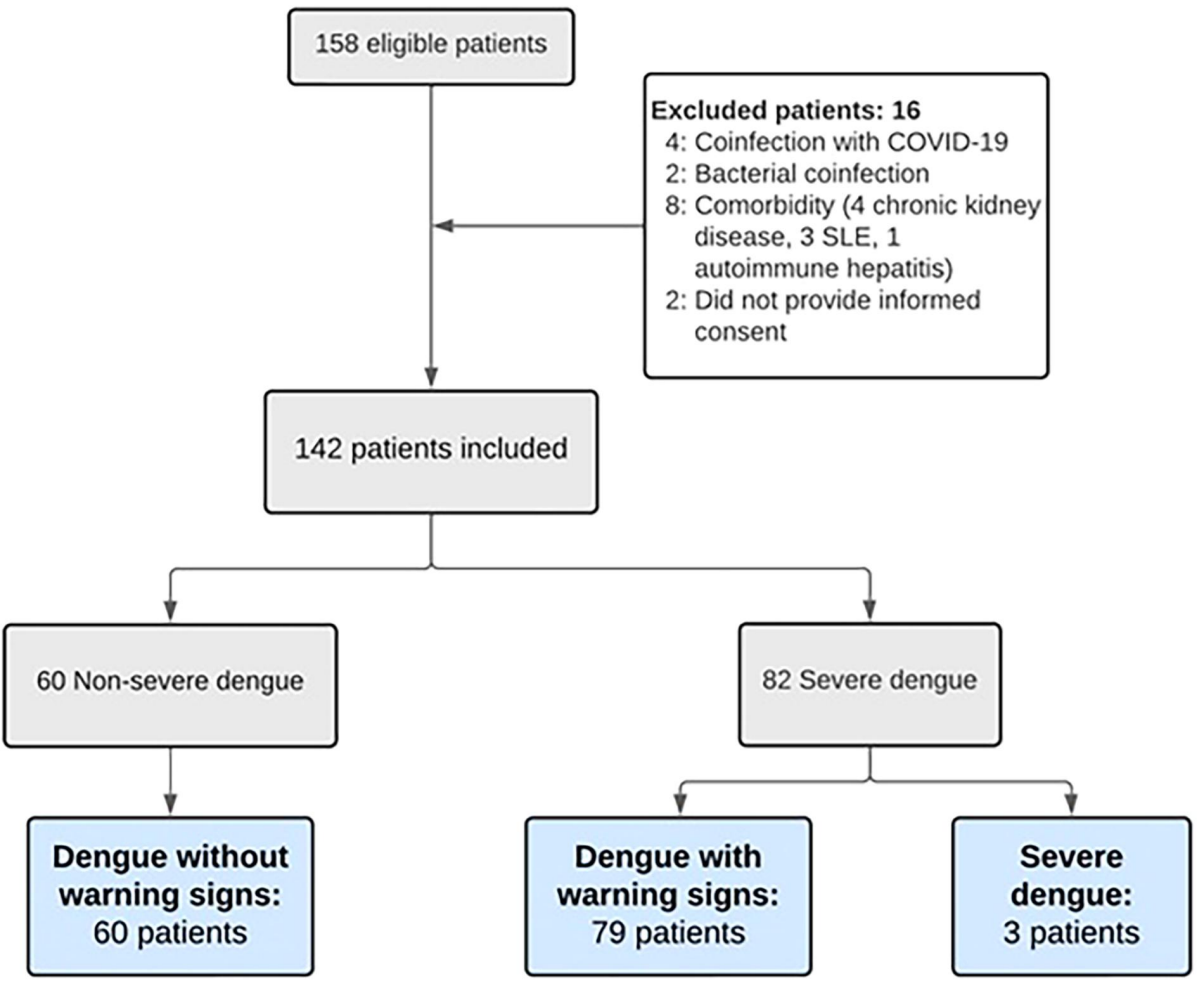
Patient flow diagram for the study.

A total of 142 patients were included in the study (Figure 1). The mean age was 9.7 ± 4.8 years, and 54.9% were male. The overall median ferritin level was 409 ng/mL (IQR: 177–1,330).

**Figure 2:**
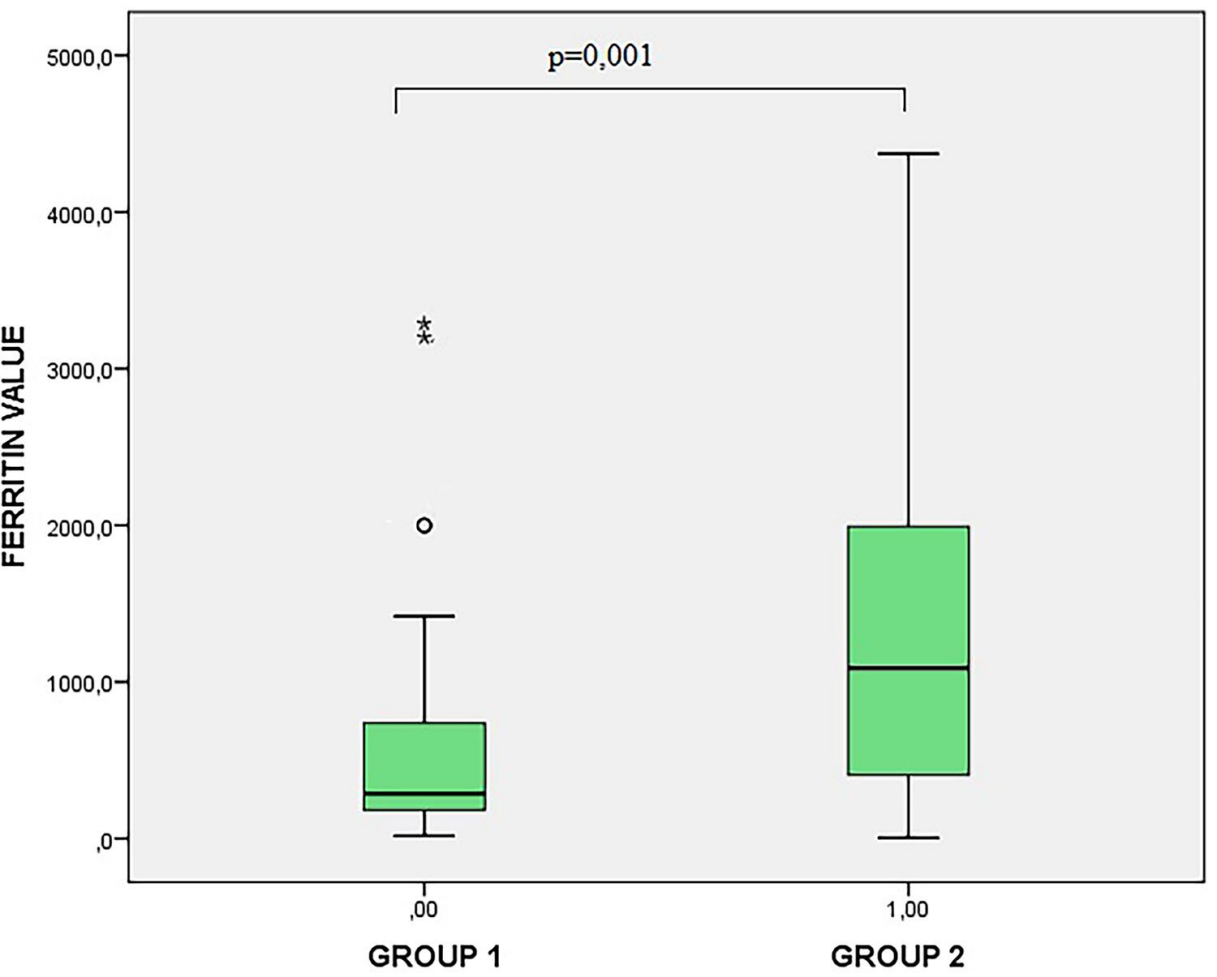
Distribution of ferritin levels across dengue severity groups.

Ferritin levels differed significantly across dengue severity categories (*p* = 0.003). Median ferritin levels were 261.5 ng/mL (IQR: 130–645.5) in patients without warning signs, 813 ng/mL (IQR: 244–1,747) in those with warning signs, and 1,052 ng/mL (IQR: 993–1,112) in patients with severe dengue. Pairwise comparisons showed significantly higher ferritin levels in patients with warning signs compared to those without (Figure 2).

**Figure 3:**
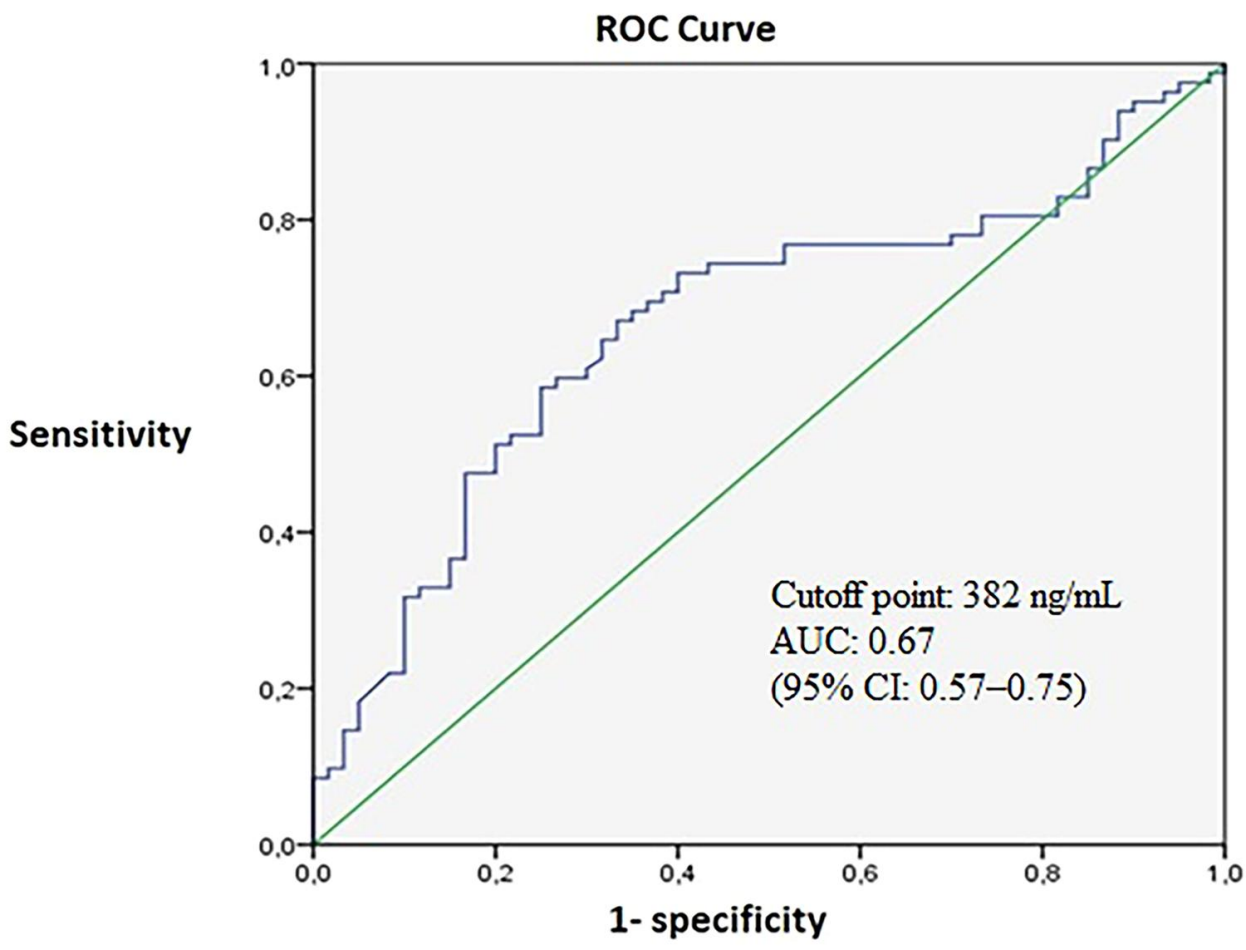
Distribution of ferritin levels across study groups.

According to the study classification, 42.2% of patients were assigned to group 1 and 57.8% to group 2. Median ferritin levels were significantly higher in group 2 compared with group 1 [841.5 ng/mL (IQR: 244–1,747) vs. 261.5 ng/mL (IQR: 130–645.5); *p* = 0.001] (Figure 3).

**Figure 4:**
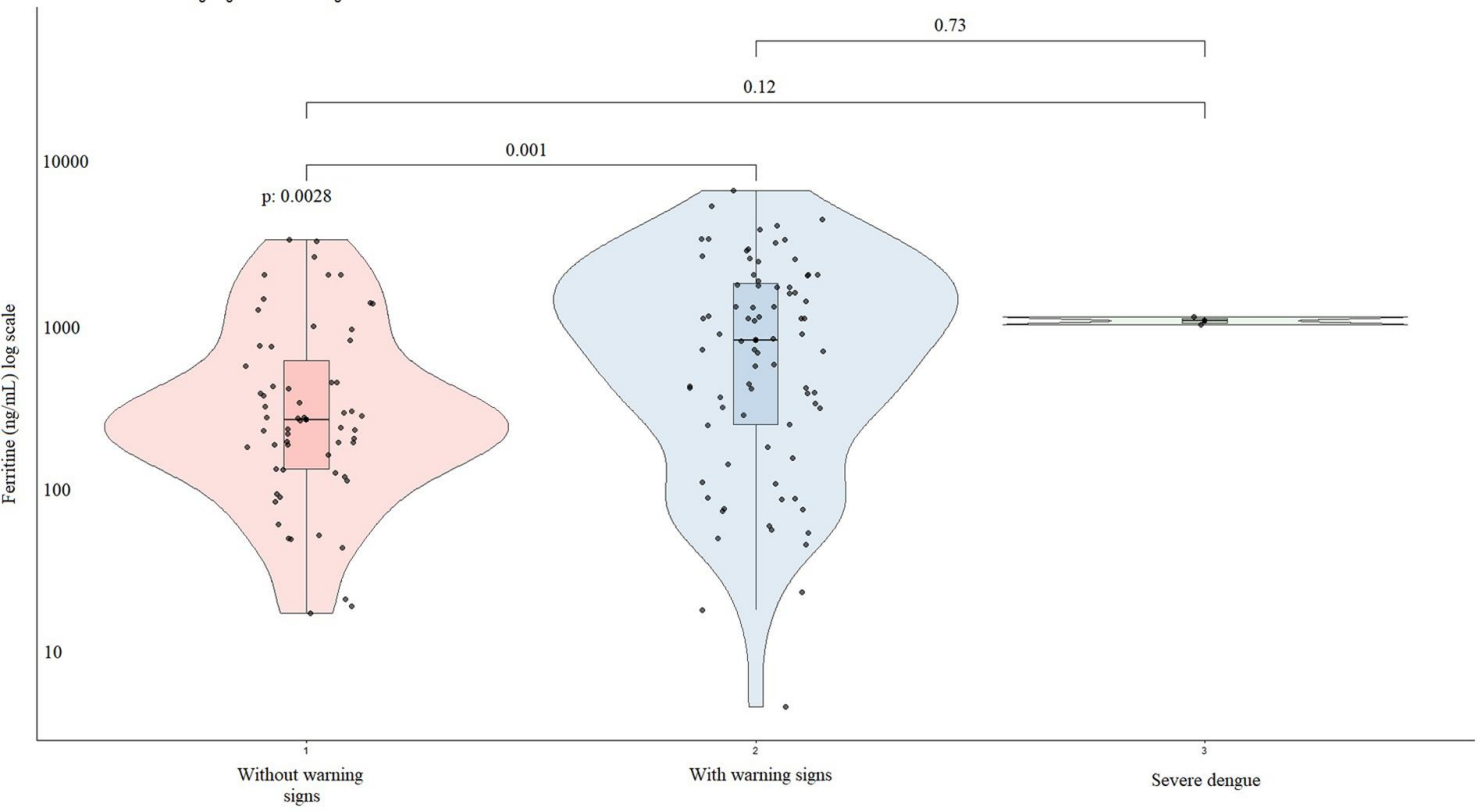
ROC curve of ferritin values to identify severe dengue.

The area under the receiver operating characteristic (ROC) curve for ferritin in predicting dengue severity was 0.67 (95% CI: 0.57–0.75). A ferritin cutoff value of 382 ng/mL yielded a sensitivity of 67%, specificity of 67%, positive predictive value (PPV) of 60%, negative predictive value (NPV) of 73%, positive likelihood ratio (LR+) of 2.0, and negative likelihood ratio (LR−) of 0.49 (Figure 4).

Ferritin levels ≥382 ng/mL were significantly associated with dengue with warning signs or severe dengue (39%) compared with non-severe dengue (14%) (*p* < 0.001; RR: 4.0; 95% CI: 2.0–8.2).

A total of 23 patients had comorbidities, including obesity (7/23), asthma (5/23), congenital heart disease (3/23), chronic non-progressive encephalopathy (2/23), and congenital hypothyroidism (1/23), among others.

Table 1 summarizes the characteristics of patients according to ferritin levels.

**Table 1:** Demographic characteristics, study groups according to ferritin cut-off point.

Variable	Total (n:142)	Ferritin <382 ng/mL (n:67)	Ferritin ≥382 ng/mL (n:75)	*p*
Age years (M ± SD)	9.7 ± 4.8	8.91 ± 4.8	10.1 ± 4.6	0.082
Sex				
Male—f, (%)	78 (55%)	36 (46%)	42 (54%)	0.78
Female—f, (%)	64 (45%)	31 (48%)	33 (52%)	
Study groups				
Dengue without warning signs f, (%)	60 (42%)	40 (67%)	20 (33%)	0.001
Dengue with warning signs—f, (%)	79 (56%)	27 (34%)	52 (66%)	
Severe Dengue—f, (%)	3 (2%)	0	3 (100%)	

M, mean; SD, standard deviation; f, frequency; %, percentage.

Among patients in group 2, the most frequent warning sign was persistent abdominal pain (62/89), followed by persistent vomiting (34/89), hemorrhagic manifestations (27/89), and altered consciousness or seizures (1/89). Fifty-three percent of patients presented with a single warning sign, while 36% had two or more. No significant association was found between ferritin levels ≥382 ng/mL and the presence of multiple warning signs (*p* = 0.16).

Ferritin levels did not differ significantly by sex (male: median 414 ng/mL; female: 404 ng/mL; *p* = 0.28) or by age group (<5 years: 223 ng/mL vs. ≥5 years: 419 ng/mL; *p* = 0.11).

The clinical characteristics of patients are shown in Table 2. No significant differences were observed in vital signs or the presence of hypotension. A total of 89 ultrasound studies were performed (Table 3). Findings suggestive of capillary leak (free fluid and/or gallbladder wall thickening) were more frequent in group 2 (24 vs. 4; *p* = 0.002; RR: 7.7; 95% CI: 2.3–24.8). Pleural effusion was identified in seven patients, all of whom had additional ultrasound findings consistent with capillary leak.

**Table 2:** Clinical characteristics and management of patients.

Characteristics	Ferritin <382 ng/mL (*n*:67)	Ferritin ≥382 ng/mL (*n*:75)	*p*	RR (95% CI)
Heart rate (M ± SD)	100.4 ± 17.8	99.9 ± 20.7	0.89	NA
Systolic pressure (M ± SD)	104.5 ± 11.2	107.8 ± 11	0.08	NA
Diastolic pressure (M ± SD)	66.3 ± 10.4	67.2 ± 9	0.56	NA
Differential pressure (M ± DE)	39.3 ± 10.2	40.5 ± 8.8	0.44	NA
Hypotension for age—f, (%)	1	2	0.62	NA
ICU admission	0	1	0.3	NA
Vasoactive infusion	1	2	0.62	NA
Fluid replacement protocol				
Yes	25 (37%)	46 (61%)	0.04	2.6 (1.3–5.2)
No	42 (63%)	29 (39%)		

M, mean; SD, standard deviation; f, frequency; %, percentage; m, median; IQR, interquartile range; RR, relative risk; NA, not applicable.

**Table 3:** Results of ultrasound studies according to ferritin cut-off point.

Ultrasound studies performed on the patients studied.	Ferritin <382 ng/mL (*n*:15)	Ferritin ≥382 ng/mL (*n*:74)	*p*	RR (95% CI)
Abnormal abdominal ultrasound—f, (%)	4 (27%)	26 (35%)	0.002	7.7 (2.3–24.8)

f, frequency; %, percentage; RR, relative risk.

The mean length of hospital stay was 3 days in both groups, with no significant difference (*p* = 0.09). The need for fluid resuscitation was more frequent in patients with ferritin ≥382 ng/mL (*p* = 0.04; RR: 2.6; 95% CI: 1.3–5.2).

In multivariable logistic regression analysis, ferritin was not independently associated with dengue severity (OR: 1.003; *p* = 0.099), and none of the included covariates reached statistical significance.

Only one patient required admission to the pediatric intensive care unit. This 12-year-old male presented with hypotension and severe cardiovascular dysfunction, requiring inotropic support and mechanical ventilation. He died 18 h after admission. His ferritin level was 993 ng/mL.

## Discussion

Hyperferritinemia and its association with disease severity have been reported in multiple studies, reflecting a hyperinflammatory response of the immune system to infection (12, 14). In our cohort, the median ferritin level was 409 ng/mL (IQR: 177–1,330), with values increasing according to clinical severity. However, these levels were lower than those reported by Goyal et al. (15), who observed a mean ferritin level of 1,469.43 ± 297 ng/mL in 70 patients, as well as in other published series.

Variability in ferritin levels across studies has been previously described. Shukla et al. (16), in a systematic review and meta-analysis, reported that the cut-off value to discriminate between non-severe and severe dengue ranged from 423 to 1,200 ng/mL. This variability may be explained by contextual factors influencing ferritin levels, such as genetic background, iron status, and socioeconomic conditions, which were not assessed in the present study.

In our analysis, ferritin levels were higher in patients with dengue with warning signs or severe dengue compared to those with non-severe disease, and elevated ferritin levels were associated with an increased risk of this composite outcome. These findings are consistent with those reported by Kiran et al. (10), where higher ferritin levels were observed in patients with severe dengue, and with studies showing that this association persists up to the fifth day of illness (11). Together, these results support the role of ferritin as a marker of systemic inflammation in dengue and other infectious diseases.

Despite this association, the discriminatory performance of ferritin was modest. The area under the curve (AUC) of 0.67 indicates fair but limited accuracy, with moderate sensitivity and specificity. Furthermore, in multivariable analysis, ferritin was not independently associated with the outcome, underscoring the multifactorial nature of dengue severity and the limited ability of a single biomarker to capture this complexity. These findings suggest that ferritin should be interpreted as an adjunctive marker rather than a standalone predictor, and its clinical utility is likely enhanced when combined with other clinical and laboratory parameters.

Although elevated ferritin levels (≥382 ng/mL) were associated with an increased risk of dengue with warning signs or severe dengue, diagnostic performance metrics—including sensitivity, specificity, Youden index, and likelihood ratios—were only moderate. This contrasts with other studies reporting higher diagnostic accuracy (11, 12), reinforcing the need to integrate ferritin with other tools to improve early identification of patients at risk of severe disease (2, 13).

Ferritin levels above the proposed cut-off were not associated with length of hospital stay, in contrast to studies reporting a moderate correlation between ferritin and hospitalization duration (17). This discrepancy may be explained by admission criteria applied to patients with non-severe dengue, such as dehydration or the presence of comorbidities, which may influence hospitalization independently of disease severity (2).

No significant differences were observed in the number of warning signs between groups, differing from findings by Soundravally et al. (12), who reported associations between elevated ferritin levels and specific warning signs such as hemorrhagic manifestations and thrombocytopenia. However, ultrasound findings suggestive of capillary leak—such as free fluid and gallbladder wall thickening—were significantly more frequent in patients with ferritin levels above the cut-off, with a 7.7-fold increased risk. These patients also required more frequent fluid resuscitation, reflecting greater clinical severity and need for intervention in accordance with current management guidelines (2). These findings support a potential association between ferritin and clinically relevant manifestations, although insufficient for independent risk stratification.

No significant differences were found in vital signs, including heart rate, systolic and diastolic blood pressure, or pulse pressure, among patients with elevated ferritin levels. This may be explained by the fact that many patients were in early stages of capillary leak. Previous studies have shown that vital signs may remain within normal ranges during early or compensated phases of dengue shock (17, 18), consistent with observations by Iramain et al. (19).

The clinical utility of serum ferritin in the emergency setting has been described in previous studies and is supported by our findings. However, the proposed cut-off value requires integration with other clinical tools to improve the identification of patients at risk of severe dengue.

Several limitations should be acknowledged. First, this was a single-center study, which may limit generalizability and requires validation in other settings. Second, the small number of patients with WHO-defined severe dengue limits subgroup-specific conclusions, and results should therefore be interpreted within the context of the composite outcome used. Third, ultrasound was performed selectively in patients with warning signs, which may introduce selection bias. Finally, variability in the timing of ferritin measurement represents an important limitation, as ferritin levels may change throughout the course of the disease. Although the mean day of sampling was similar between groups, residual temporal variability cannot be excluded and may have influenced the observed differences.

Future studies with standardized timing of biomarker measurement are needed to better define the temporal dynamics and diagnostic performance of ferritin in dengue.

## Conclusion

Higher ferritin levels were observed in patients with dengue requiring clinical intervention, including hospitalization or fluid resuscitation. Although a cutoff value of ≥382 ng/mL was associated with dengue with warning signs or severe dengue, its discriminatory performance was limited. These findings suggest that ferritin may serve as an adjunctive biomarker but should not be used as a standalone predictor of disease severity in pediatric patients with dengue.

## Data Availability

The raw data supporting the conclusions of this article will be made available by the authors, without undue reservation.

## References

[1] Fonseca SNS. Changing epidemiology of dengue fever in children in South America. Curr Opin Pediatr. (2023) 35:147–54. 10.1097/MOP.0000000000001220 36715049

[2] Pan American Health Organization. Guidelines for the clinical diagnosis and treatment of dengue, chikungunya, and Zika (2022). 10.37774/9789275124871

[3] General Directorate of Health Surveillance—MSP and BS. Epidemiological alert for dengue (2023). Available online at: https://www.mspbs.gov.py/portal/28924/alerta-epidemiologica-por-dengue.html (Accessed November 15, 2025).

[4] Institute of Tropical Medicine. Dengue and Chikungunya Epidemic Management Guide (2023). Available online at: https://www.mspbs.gov.py/dependencias/imt/adjunto/29ea36-GuiadeChikungunyayDengue.pdf (Accessed November 15, 2025).

[5] Tayal A Kabra SK Lodha R. Management of dengue: an updated review. Indian J Pediatr. (2023) 90(2):168–77. 10.1007/s12098-022-04394-8 36574088 PMC9793358

[6] Pavlicich V. Dengue: review and experience in pediatrics. Arch Pediatr Urug. (2016) 87(2):143–56. Available online at: http://www.scielo.edu.uy/scielo.php?script=sci_arttext&pid=S1688-12492016000200011

[7] CDC. Management of dengue cases (2024). Available online at: https://www.cdc.gov/dengue/media/pdfs/2024/06/2024-Dengue-Clinical-Management-Pocket-Guide-ES.pdf (Accessed November 15, 2025).

[8] Kernan KF Carcillo JA. Hyperferritinemia and inflammation. Int Immunol. (2017) 29(9):401–9. 10.1093/intimm/dxx031 28541437 PMC5890889

[9] Plays M Müller S Rodriguez R. Chemistry and Biology of Ferritin. Vol. 13. Metallomics: Oxford University Press (2021). 10.1093/mtomcs/mfab021 PMC8083198 33881539

[10] Kiran G Yerra S. Diagnostic value of serum ferritin as an indicator of disease severity in children with dengue infection. Natl J Physiol Pharm Pharmacol. (2022) 12(12):1. 10.5455/njppp.2022.12.05195202208052022

[11] Mahendranath K Langade RA. To find out role of serum ferritin levels as an early predictor of severity of dengue-an observational study. Int J Health Sci (Qassim). (2022) 6:4119–28. 10.53730/ijhs.v6nS1.5750

[12] Soundravally R Agieshkumar B Daisy M Sherin J Cleetus CC. Ferritin levels predict severe dengue. Infection. (2015) 43(1):13–9. 10.1007/s15010-014-0683-4 25354733

[13] Reddy AD Musali SR. A study on predictors of severe dengue in pediatric patients in a tertiary care hospital in telangana. Indian J Child Health (Bhopal). (2021) 8(4):162–5. 10.32677/IJCH.2021.v08.i04.006

[14] Moras E Achappa B Murlimanju BV Raj GMN Holla R Madi D Early diagnostic markers in predicting the severity of dengue disease. 3 Biotech. (2022) 12(10):268. 10.1007/s13205-022-03334-9 36091089 PMC9461388

[15] Goyal PK Hissaria K Shekhawat C. Role of Serum ferritin as a predictor of dengue severity: a prospective observational study from India. Cureus. (2024) 16:e63503. 10.7759/cureus.63503 39081444 PMC11288214

[16] Shukla S Jadhav SM Gurav YK Parashar D Alagarasu K. Serum ferritin level as a prognostic biomarker for predicting dengue disease severity: a systematic review and meta-analysis. Rev Med Virol. (2023) 33:e2468. 10.1002/rmv.2468 37347209

[17] Durán CA Lanza TM Plata JA Durán C. Historical and epidemiological aspects of the pathophysiology and diagnosis of dengue. Rev Med Hondur. (2010) 78:136–41.

[18] Kularatne SA. Dengue fever. Br Med J. (2015) 351:h4661. 10.1136/bmj.h4661 26374064

[19] Iramain R Jara A Cardozo L Bogado N Morinigo R. Treatment of dengue shock in a pediatric emergency unit. Pediatr (Asunción). (2013) 40(1):11–8. Available online at: http://scielo.iics.una.py/scielo.php?script=sci_arttext&pid=S1683-98032013000100002&lng=en

